# Bridging the urban–rural divide: digital literacy as a catalyst for enhancing physical exercise participation in China

**DOI:** 10.3389/fpubh.2025.1630850

**Published:** 2025-09-23

**Authors:** Wangjie Li, Zhan Chen

**Affiliations:** ^1^School of Physical Education, Nanyang Normal University, Nanyang, China; ^2^School of Sports Science and Physical Education, Nanjing Normal University, Nanjing, China

**Keywords:** digital literacy, physical exercise, urban–rural disparity, health behavior, health equity, China Family Panel Studies, digital empowerment

## Abstract

**Background:**

Rapid societal digitalization is transforming the determinants of health behaviors worldwide. Digital literacy—defined as the ability to effectively access, evaluate, and utilize digital information—appears crucial in promoting physical activity and mitigating health disparities. However, empirical evidence on its influence within the context of China’s pronounced urban–rural divide remains limited.

**Methods:**

Utilizing data from the 2022 China Family Panel Studies (*N* = 18,336), this study applied logit, OLS, and IV-2SLS models to examine digital literacy’s effect on exercise frequency and duration. Heterogeneity was assessed via subgroup analyses and interaction tests.

**Results:**

Elevated digital literacy demonstrated significant positive correlations with exercise frequency (*p* < 0.01) and duration (*p* < 0.01). Crucially, this relationship exhibited marked urban–rural heterogeneity, with substantially stronger effects observed in rural populations. Baseline regression analyses quantified these patterns: a one-unit increase in digital literacy corresponded to a 1.72-unit rise in weekly exercise frequency and a 26.19-min extension in per-session duration across the full sample (both *p* < 0.01). When stratified by residence, rural participants showed significantly greater responsiveness—digital literacy increments yielded increases of 2.05 frequency units and 29.34 min per session, versus 0.99 units and 19.23 min among urban counterparts. Formal interaction tests confirmed this divergence, revealing rural advantages of +1.76 frequency units (95% CI: 1.35–2.17; *p* < 0.01) and +10.88 min per session (95% CI: 4.61–17.16; *p* < 0.01). All findings persisted through instrumental variable and sensitivity analyses.

**Conclusion:**

Digital literacy critically enables health-promoting behaviors, particularly for rural residents facing structural resource constraints. Enhancing digital competencies may narrow urban–rural gaps in health behaviors and advance health equity. Policymakers should prioritize rural digital infrastructure and digital skills training to fully harness digital empowerment for public health.

## 1 Introduction

The pervasive integration of digital networks has established digitalization as an innovative pathway for enhancing the quality and inclusivity of national physical fitness initiatives (1–3). Increasingly, digital technologies serve as valuable tools for optimizing sports infrastructure utilization, advancing national fitness policies, and mitigating physical activity disparities (1, 4). However, recent evidence suggests that expanded internet coverage does not inherently translate into increased physical activity (5); paradoxically, it may correlate with sedentary behaviors, elevated obesity rates, and heightened chronic disease risks (6). This phenomenon primarily stems from excessive screen time (7), intensive digital media engagement (4), and online gaming addiction (8).

An emerging scholarly consensus highlights digital literacy—defined as the competency to effectively access, evaluate, and utilize digital information (9)—as a critical determinant. Individuals with advanced digital literacy demonstrate superior capacity to leverage technologies for acquiring health knowledge, engaging in health-promoting interactions, and adopting beneficial behaviors such as regular exercise and balanced nutrition (10, 11). Within physical activity contexts, while smartphone applications, wearable devices, and online fitness communities reduce participation barries, their efficacy remains contingent upon users’ digital literacy (12). Consequently, digital literacy has emerged as a significant social determinant of health behaviors (13, 14).

Nevertheless, rural China experiences inequitable distribution of digitalization’s benefits. Structural constraints—including economic underdevelopment, limited education, and digital skill deficits—severely restrict residents’ capacity to harness technological advantages (15). Persistent urban–rural divides in digital infrastructure, competencies, and resource access exacerbate health inequalities (9, 16, 17). Although national policies such as the “Digital Village Strategy” (18) and “Internet Poverty Alleviation Action Plan” have expanded rural 5G coverage to over 90% of villages (19), field studies consistently reveal enduring disparities across three dimensions: markedly reduced availability and suitability of digital resources (supply gap), limited affordability of devices/services (affordability gap), and weaker operational skills and motivation (usage gap) (16, 20, 21). These gaps collectively restrict access to online fitness platforms (22) and exacerbate physical activity disparities (23). Revealing a critical disconnect between infrastructure investment and behavioral outcomes—a nexus underexplored in extant research.

**Figure 1:**
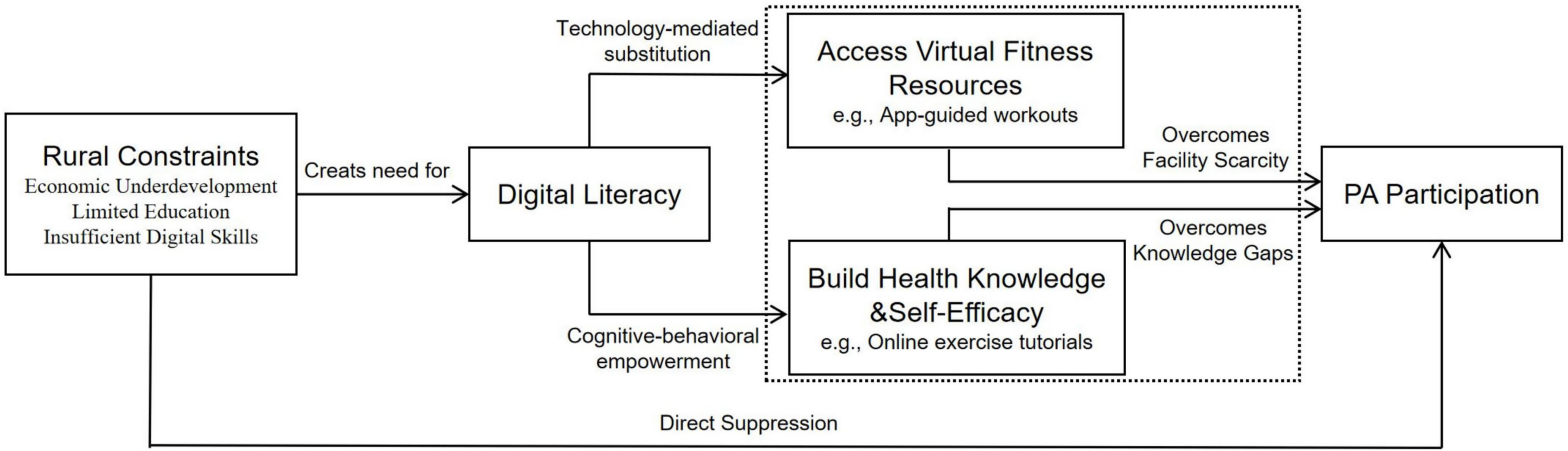
Dual-path compensation model of digital literacy in rural exercise promotion.

This digital marginalization intensifies urban–rural divides in health information access, healthcare utilization, and crucially, exercise participation (15). Precisely due to these constraints, digital literacy may function as a compensatory mechanism, enabling rural residents to circumvent structural barriers through alternative digital pathways. We posit two synergistic compensatory routes: (i) Technology-mediated compensation: Using fitness apps/wearables to access exercise resources (12, 22), effectively substituting for scarce physical facilities; (ii) Cognitive-behavioral compensation: Enhancing health knowledge and self-efficacy through health literacy (24) and social cognitive theory (25), counteracting educational limitations. These mechanisms are integrated in our conceptual framework (Figure 1), which posits that rural contexts amplify digital literacy’s compensatory potency.

Prior research has predominantly examined digital health literacy in clinical settings (e.g., medical information-seeking, chronic disease management) (13, 14), with relatively sparse empirical attention to physical activity behaviors. Crucially, Few studies directly investigate digital literacy’s influence on exercise patterns, and none systematically compare urban–rural differentials. Rural residents’ limited access to sports facilities and professional guidance heightens the theoretical importance of digital solutions (12)—yet robust empirical validation remains lacking. Addressing this gap, our study leverages nationally representative data from the 2022 China Family Panel Studies (CFPS) to:

(i) To what extent does digital literacy quantitatively influence the frequency and duration of physical activity participation?
(ii) How does this relationship differentially manifest between urban and rural populations, when rigorously controlling for endogeneity through interaction terms and instrumental variable (IV-2SLS) approaches?
(iii) What evidence-based policy interventions can be derived to align with China’s rural digital empowerment initiatives for bridging the physical activity divide?

## 2 Materials and methods

### 2.1 Data source, study design, and ethics

This study utilizes data from the 2022 wave of the China Family Panel Studies (CFPS), a nationally representative longitudinal survey conducted by Peking University’s Institute of Social Science Survey (ISSS). The CFPS employs a multi-stage probability-proportional-to-size (PPS) sampling design with implicit stratification, encompassing 25 provincial-level administrative units across mainland China. This design systematically collects micro-level data on demographic characteristics, socioeconomic status, and health behaviors.

To construct the analytical sample, individual and household questionnaires were merged, retaining respondents who reported both digital technology use and physical exercise participation while excluding cases with missing values for key variables, yielding a final sample of 18,336 individuals. This sample size satisfies the criterion for complex econometric modeling, ensuring statistical power. The probability sampling framework guarantees national representativeness, and standardized instruments enhance measurement validity. As a secondary analysis of de-identified public data (CFPS-2022), this study was exempt from additional ethics review per institutional guidelines. Primary data collection received approval from Peking University’s IRB (IRB00001052-14010) (26), with written informed consent obtained from all participants.

### 2.2 Variables

#### 2.2.1 Physical exercise participation

This study constructs a multidimensional variable system to explore the relationship between digital literacy and physical exercise participation. The dependent variable—individual physical exercise participation—is measured across three dimensions: frequency, duration, and general participation, based on behavioral reports from the CFPS questionnaire over the past 12 months. Frequency as an ordinal variable (0 = never; 1 = <1/month; 2 = ≥1/month but <1/week; 3 = 1–2/week; 4 = 3–4/week; 5 = ≥5/week; 6 = daily; 7 = ≥2/day). Duration as a continuous measure (minutes per session). General participation as a binary indicator (0 = no participation; 1 = ≥1 session annually).

#### 2.2.2 Digital literacy

Drawing upon established methodologies (27, 28), digital literacy was quantified via a composite index derived from 12 items across five domains (digital usage, learning, social engagement, work applications, and daily life integration). Each item was normalized, and weights were assigned using Shannon entropy to objectively reflect discriminative power (Appendix A). The result standardized index ranges from 0 (lowest proficiency) to 1 (highest proficiency). Table 1 provides a breakdown of the digital literacy index, including its dimensions, measurement items, scoring, and the weights assigned by the entropy method.

**Table 1:** Measurement framework of digital literacy indicators.

Dimension	Measurement items	Scoring	Weight
Digital usage	Internet use via mobile and computer devices	Combined duration of mobile and computer internet usage	0.097
Digital learning digital social	Importance of internet for learning	Importance rated 1–5, very important = 5, very unimportant = 1	0.042
	Online learning behavior	Daily online learning = 2, online learning = 1, no = 0	0.200
	Importance of internet for family and friend contact	Importance rated 1–5, very important = 5, very unimportant = 1	0.038
	Use of WeChat	Yes = 1; No = 0	0.039
	Frequency of sharing on WeChat moments	Scale from 1 (never) to 7 (almost daily)	0.113
Digital work	Importance of internet for work	Importance rated 1–5, very important = 5, very unimportant = 1	0.045
Digital life	Importance of internet for daily life	Importance rated 1–5, very important = 5, very unimportant = 1	0.045
	Importance of internet for leisure and entertainment	Importance rated 1–5, very important = 5, very unimportant = 1	0.043
	Online shopping	Daily shopping = 2, occasional shopping = 1, no = 0	0.119
	Watching short videos	Daily watching = 2, occasional watching = 1, no = 0	0.015
	Online gaming	Daily gaming = 2, occasional gaming = 1, no = 0	0.204

#### 2.2.3 Control variables

Multilevel confounders were incorporated to mitigate omitted-variable bias. Individual-level controls included sociodemographic attributes (household registration type, gender, age), socioeconomic status (education years, marital status, logarithmic family income), health capital (self-rated health, chronic disease status) (29). And subjective social status is included because lower perceived social rank is associated with reduced physical exercise participation and poorer health behaviors, even after adjusting for objective socioeconomic indicators (30, 31). Evidence from the Chinese aging population further indicates that subjective social status influences exercise engagement through social trust (32), underscoring its relevance in the Chinese context. At the regional level, fixed effects control for spatial heterogeneity arising from regional developmental differences (33). Operational definitions and descriptive statistics are detailed in Table 2.

**Table 2:** Descriptive statistics for key variables.

Continuous variables	Meaning	Values	Mean	SD	Min	Max
Exercise frequency	Frequency of physical exercise participation	0 (never) to 7 (twice daily)	1.829	2.376	0	7
Exercise duration	“On average, how many minutes do you exercise each time (1–300)?”	Minutes per session	23.335	34.993	0	270
Exercise participation	Participation in physical exercise (dichotomous)	0 (never), 1 (any)	0.437	0.496	0	1
Digital literacy	Entropy-weighted index (12 items)	Standardized index (0–1)	0.315	0.186	0.012	0.987
Urban	Household registration type	0 (rural), 1 (urban)	0.298	0.457	0	1
Gender	Respondent’s gender	0 (female), 1 (male)	0.503	0.500	0	1
Age	Respondent’s age in years	Years	45.223	16.764	16	97
Years of education	Completed years of formal education	Years of formal education	9.440	4.686	0	23
Ln household income	Natural log of per capita household income (yuan)(CNY)	Log of per capita household income	10.005	1.077	0	15.745
Subjective social status	On a scale of 1–5, where do you place your social status locally?	1 (very low) to 5 (very high)	3.009	1.053	1	5
Self-rated health	Overall health	1 (worst) to 5 (best)	3.142	1.168	1	5
Chronic disease	Chronic disease diagnosis (past 6 months)	0 (no), 1 (yes)	0.156	0.363	0	1

Categorical variable			N	%		
Marital status	Current marital status					
		Unmarried	3,309	18.02%		
		Married	13,713	74.67%		
		Cohabiting	76	0.41%		
		Divorced	496	2.70%		
		Widowed	772	4.20%		
Region	Geographic region					
		East	7,725	42.06%		
		Central	5,540	30.16%		
		West	5,101	27.77%		

Continuous variables are reported as Mean ± SD, together with N, Min, and Max. Binary variables are reported as Mean ± SD where the mean equals the proportion. All statistics are based on the analytic sample (*N* = 18,336).

### 2.3 Methodology specification

The baseline model assessed digital literacy’s effect on exercise outcomes, as shown in Equation (1):


(1) $$Y_{\mathrm{hi}}=\alpha_{0}+\alpha_{1}{\mathrm{DL}}_{\mathrm{hi}}+\alpha_{2}{\text{Control}}_{\mathrm{hi}}+\varepsilon_{\mathrm{hi}}$$

where *h* denotes urban/rural subgroup, *i* the individual, *Y_hi_* the exercise outcomes (frequency/duration/participation), and *DL_hi_* the digital literacy score, *Control_hi_* is the vector of control variables, where α_0_ is the intercept; α_1_ measures the average marginal effect of Digital Literacy (DL); α_2_ is a vector of coefficients for the control variables, and *ε_hi_* is the error term.

To explicitly test urban–rural differentials, an interaction model was specified in Equation (2):


(2) $$Y_{i}=\alpha_{0}+\alpha_{1}{\mathrm{DL}}_{i}+\alpha_{2}{\text{Urban}}_{i}+\beta {\mathrm{DL}}_{i}\times {\text{Urban}}_{i}+\alpha_{3}{\text{Control}}_{i}+\varepsilon_{i}$$

A significantly negative *β* coefficient would indicate among rural residents, suggesting gap-narrowing potential.

### 2.4 Statistical analysis

All analyses were conducted in Stata 17.0. Descriptive statistics (means ± SD for continuous variables; proportions for categorical variables) and urban–rural comparisons (*t*-tests/χ^2^) were first computed. The digital literacy index was constructed using entropy weighting (Appendix A). Baseline regressions employed OLS for frequency/duration outcomes and logit for binary participation, with province-clustered robust standard errors. To address potential endogeneity, instrumental variable estimation (IV-2SLS) utilized village-level internet access rates as an instrument, subject to diagnostic tests: first-stage F-statistic >10 [relevance threshold (34)], Kleibergen-Paap LM test rejecting under-identification (*p* < 0.01), and Cragg-Donald/Kleibergen-Paap Wald statistics exceeding the Stock-Yogo 10% critical value (16.38). Sensitivity analyses included: (1) restricting to working-age adults (18–59 years), (2) excluding centrally administered municipalities, and (3) substituting binary participation for frequency/duration. Statistical significance was defined as two-tailed *p* < 0.05.

## 3 Results

### 3.1 Descriptive statistics

Substantial urban–rural disparities in physical exercise participation and socioeconomic characteristics are evident in Table 3 (all *p* < 0.01). Urban residents reported higher exercise frequency (*M* = 2.61, SD = 2.50), corresponding to approximately weekly sessions, whereas rural residents averaged significantly lower frequency (*M* = 1.50, SD = 2.24), indicating participation below weekly levels (Table 3). Similarly, urban participants engaged in longer exercise durations per session (*M* = 34.0 min, SD = 37.9) compared to rural counterparts (*M* = 18.8 min, SD = 32.7). The proportion engaging in any exercise was markedly higher in urban (60.8%) versus rural (36.5%) areas, confirming pronounced behavioral inequities.

**Table 3:** Urban–rural differences in key variables.

Variable	Rural (*N* = 12,892)	Urban (*N* = 5,474)	MeanDiff	Effect size	*p*-value	Effect size type
Continuous variables	(Mean ± SD)	(Mean ± SD)	(Urban–Rural)	[95% CI]		
Exercise frequency	1.496 ± 2.241	2.613 ± 2.498	−1.117	−0.481 [−0.449, −0.513]	<0.01	Cohen’s d
Exercise duration	18.795 ± 32.659	34.028 ± 37.866	−15.233	−0.444 [−0.412, −0.476]	<0.01	Cohen’s d
Digital literacy	0.293 ± 0.181	0.368 ± 0.185	−0.075	−0.411 [−0.379, −0.443]	<0.01	Cohen’s d
Age	44.847 ± 16.681	46.110 ± 16.925	−1.263	−0.075 [−0.044, −0.107]	<0.01	Cohen’s d
Years of education	8.459 ± 4.617	11.752 ± 3.980	−3.294	−0.742 [−0.710, −0.775]	<0.01	Cohen’s d
Ln household income	9.794 ± 1.045	10.503 ± 0.985	−0.709	−0.690 [−0.658, −0.722]	<0.01	Cohen’s d
Subjective social status	3.048 ± 1.093	2.917 ± 0.948	0.131	0.125 [0.156, 0.093]	<0.01	Cohen’s d
Self-rated health	3.163 ± 1.211	3.093 ± 1.059	0.069	0.059 [0.091, 0.028]	<0.01	Cohen’s d

Binary variables	Rural (N, %)	Urban (N, %)				
Exercise participation	4,706 (36.5%)	3,351 (61.2%)		0.243 [0.227, 0.258]	**<0.01**	RD
Gender (male)	6,498 (50.4%)	2,737 (50.0%)		0.004 [−0.020, 0.012]	0.597	RD
Chronic disease	1,895 (14.7%)	958 (17.5%)		0.028 [0.016, 0.040]	**<0.01**	RD

Categorical variables	(N, %)	(N, %)	Chi-square			
Marital status			41.336	V = 0.047	**<0.01**	Cramer’s V
Unmarried	2,365 (18.34%)	944 (17.25%)				
Married	9,634 (74.73%)	4,079 (74.52%)				
Cohabiting	46 (0.36%)	30 (0.55%)				
Divorced	289 (2.24%)	207 (3.78%)				
Widowed	558 (4.33%)	214 (3.91%)				
Region			319.509	*V* = 0.132	**<0.01**	Cramer’s V
East	5,106 (39.61%)	2,619 (47.84%)				
Central	3,710 (28.78%)	1,830 (33.43%)				
West	4,076 (31.62%)	1,025 (18.72%)				

*Effect size interpretation:* Cohen’s d: negligible (<0.2), small (0.2–0.49), medium (0.5–0.79), large (≥0.8); Risk Difference (RD): Absolute difference in proportions (Urban - Rural); Cramer’s V: weak (0.1–0.29), moderate (0.3–0.49), strong (≥0.5). *Sample size consideration:* The rural-to-urban ratio (2.35:1) increases power to detect smaller effects in rural comparisons, but effect size metrics provide sample-size-independent measures of practical significance. *p* < 0.01, two-tailed tests. Bold values indicate statistical significance.

Concomitant disparities emerged in digital literacy and socioeconomic indicators: urban residents demonstrated significantly higher digital literacy scores, educational attainment, and household income (Table 3). These differentials may compound existing health behavior inequalities.

**Figure 2:**
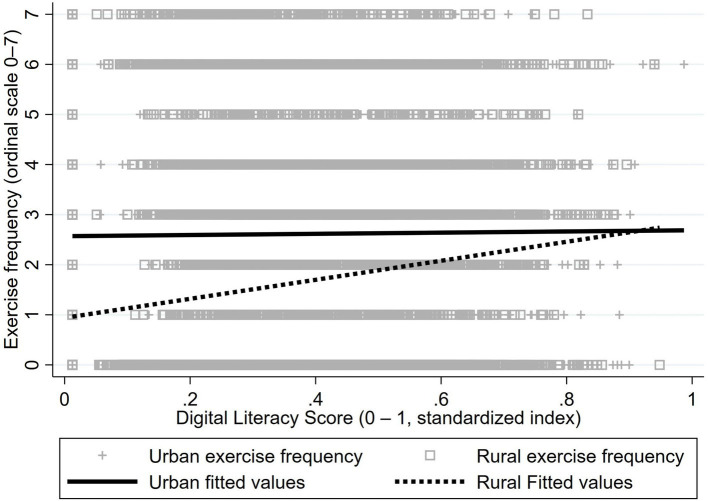
Linear fit comparison of urban–rural digital literacy and exercise frequency. Urban line: y = 2.57 + 0.12 × DL (*r* = 0.01, *R*^2^ = 0.0001, *P* = 0.518); Rural line: y = 0.94 + 1.90 × DL (*r* = 0.15, *R*^2^ = 0.0235, *p* < 0.01).

**Figure 3:**
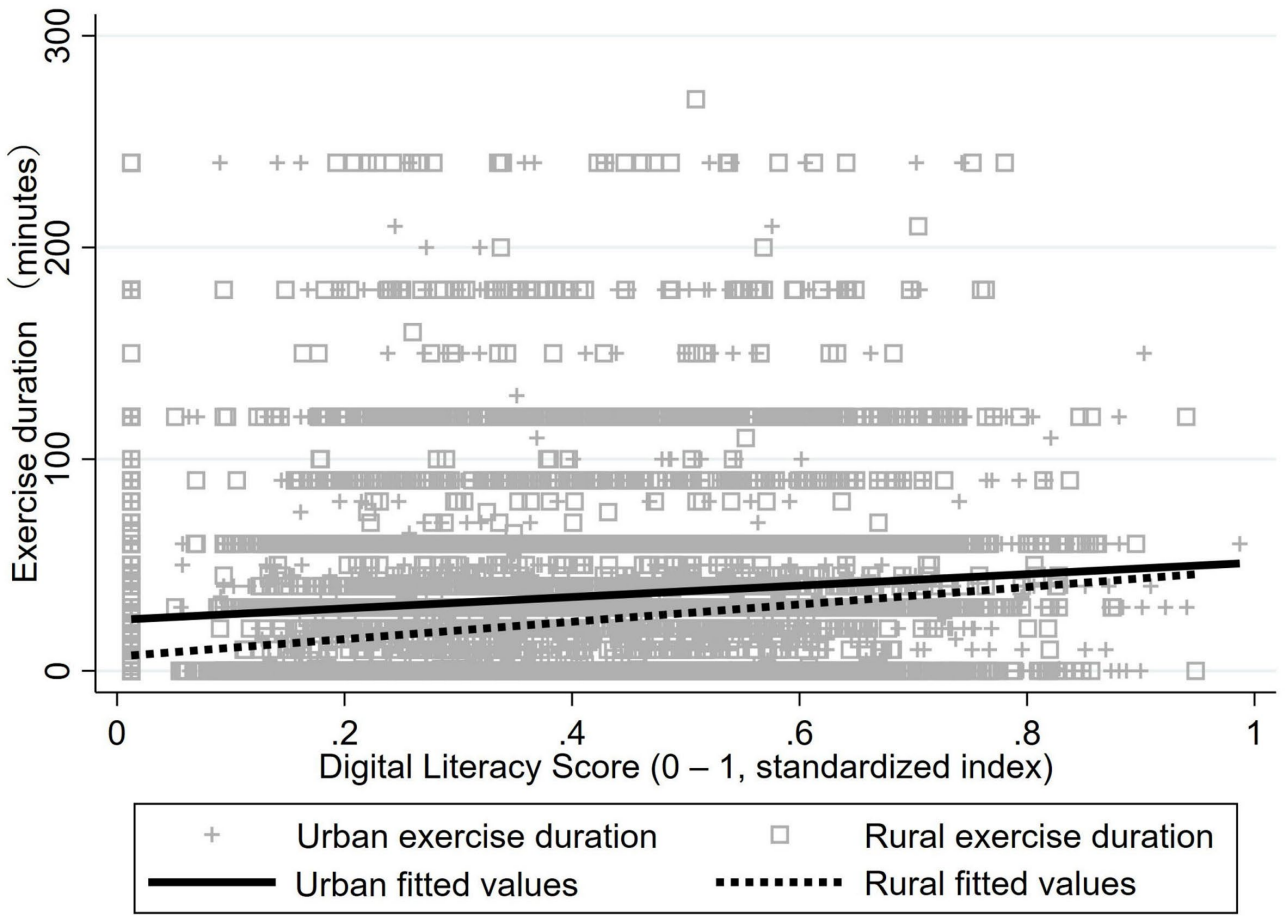
Linear fit comparison of urban–rural digital literacy and exercise duration. Urban line: y = 24.10 + 26.98 × DL (*r* = 0.13, *R*^2^ = 0.0173, *P* < 0.01); Rural line: y = 6.79 + 40.97 × DL (*r* = 0.23, *R*^2^ = 0.0517, *P* < 0.01).

Critically, linear fits (Figures 2, 3) revealed positive associations between digital literacy and exercise metrics in both groups, with steeper slopes among rural residents. Although urban fitted lines remained elevated, digital literacy increments correlated with larger physical activity gains in rural populations—particularly for exercise frequency (Figure 2). This pattern suggests digital literacy’s potential to attenuate urban–rural participation gaps.

These findings imply that despite structural divides in digital infrastructure, sports facilities, and human capital, individual digital literacy may mitigate resource limitations and reconfigure health behaviors, especially in rural contexts. Digital competence thus emerges as a pivotal lever for bridging exercise participation inequities and advancing rural health governance.

### 3.2 Baseline regression: digital literacy and exercise participation disparities

Table 4 presents baseline regression results quantifying digital literacy’s influence on exercise outcomes. Across all specifications, digital literacy demonstrated a statistically significant positive association with exercise frequency and duration (*p* < 0.01) for the full sample and urban/rural subsamples, robustly addressing Research Question (i). For the full sample, a one-unit increase in digital literacy corresponded to a 1.72-unit rise in exercise frequency (95% CI: 1.45–1.99) and a 26.19-min extension in session duration (95% CI: 22.22–30.16). Substantial urban–rural differentials emerged: rural residents exhibited greater responsiveness with 2.05-unit (95% CI: 1.73–2.37) and 29.34-min (95% CI: 24.61–34.07) gains per unit digital literacy increase, whereas urban counterparts showed comparatively smaller improvements (0.99 units, 95% CI: 0.51–1.46; 19.23 min, 95% CI: 12.00–26.45).

**Table 4:** Baseline regression results of digital literacy on urban–rural disparity in exercise participation.

Models	Exercise frequency				Exercise duration (minutes)			
Variables	Full sample	Urban	Rural	Full sample	Full sample	Urban	Rural	Full sample
Digital literacy	1.718^***^	0.988^***^	2.050^***^	2.337^***^	26.19^***^	19.23^***^	29.34^***^	30.02^***^
	(0.137)	(0.243)	(0.165)	(0.151)	(2.024)	(3.685)	(2.412)	(2.255)
Urban	0.692^***^			1.303^***^	9.453^***^			13.24^***^
	(0.042)			(0.091)	(0.636)			(1.218)
Interaction term				−1.759^***^				−10.88^***^
				(0.211)				(3.201)
Controls	Yes	Yes	Yes	Yes	Yes	Yes	Yes	Yes
Regional effects	Yes	Yes	Yes	Yes	Yes	Yes	Yes	Yes
_cons	−1.672^***^	−3.044^***^	−0.647^***^	−1.816^***^	−19.17^***^	−37.58^***^	−7.579^**^	−20.06^***^
	(0.197)	(0.387)	(0.235)	(0.198)	(2.902)	(5.934)	(3.397)	(2.925)
N	18,366	5,474	12,892	18,366	18,366	5,474	12,892	18,366
*r* ^2^	0.093	0.082	0.049	0.098	0.102	0.064	0.073	0.103

^***^means significant at the 1% confidence level; ^**^means significant at the 5% confidencelevel. “Interaction Term” is the interaction between digital literacy and rural residence. All models include the full set of controls and regional fixed effects. Robust standard error in (). The same below.

Critically, interaction models incorporating the digital literacy × urban–rural term confirmed these disparities. The significantly negative coefficients (*β* = −1.76 for frequency, *β* = −10.88 for duration; *p* < 0.01) indicate larger marginal effects in rural populations. Quantitatively, rural residents gained an additional 1.76 frequency units (95% CI: 1.35–2.17) and 10.88 min per session (95% CI: 4.61–17.16) relative to urban residents per unit digital literacy increment. These findings directly address Research Question (ii) by quantifying digital literacy’s gap-narrowing potential and provide empirical foundations for targeted rural digital empowerment policies (Research Question iii).

### 3.3 Endogeneity and robustness checks

#### 3.3.1 Endogeneity tests

To address potential endogeneity concerns, instrumental variable (IV) estimation was employed using the village-level digital access rate as an instrument. This variable captures local digital infrastructure that theoretically influences individual digital literacy but remains uncorrelated with exercise behavior, satisfying relevance and exogeneity conditions (Table 5).

**Table 5:** Endogeneity test results.

Models	Urban			Rural			Full sample		
Variables	Digital literacy	Exercise frequency	Exercise duration	Digital literacy	Exercise frequency	Exercise duration	Digital literacy	Exercise frequency	Exercise duration
Village internet access rate	0.042^***^			0.066^***^			0.031^***^		
	(0.014)			(0.009)			(0.008)		
Digital literacy		14.46^*^	310.8^**^		5.900^**^	109.4^***^		11.11^***^	208.9^***^
		(7.783)	(135.2)		(2.836)	(37.984)		(3.392)	(49.18)
Interaction term							1.338^***^	−6.941^***^	−119.0^***^
							(0.008)	(2.119)	(30.835)
First-stage F statistic	8.73^***^			52.20^***^			16.41^***^		
Kleibergen-Paap LM test	7.71^***^	7.666^***^	7.666^***^	39.34^***^	39.511^***^	39.511	14.35^***^	37.872^***^	37.872^***^
Cragg-Donald Wald F	12.02	11.979	11.979	72.59	73.089	73.089	26.24	64.925	64.925
KP Wald F	8.73	8.676	8.676	52.20	52.491	52.491	16.41	46.196	46.196
Stock-Yogo 10% threshold	16.38	16.38	16.38	16.38	16.38	16.38	16.38	16.38	16.38
Controls	Yes	Yes	Yes	Yes	Yes	Yes	Yes	Yes	Yes
Regional effects	Yes	Yes	Yes	Yes	Yes	Yes	Yes	Yes	Yes
N	3,591	3,591	3,591	9,967	9,967	9,967	13,558	13,558	13,558

*p < 0.1; **p < 0.05; ***p < 0.01.

First-stage results confirmed strong instrument relevance: village digital access significantly predicted individual digital literacy across all samples (*p* < 0.01). Diagnostic tests indicated robust instrument strength for rural (*F* = 52.20) and full samples (*F* = 16.41), exceeding the *F* > 10 threshold and Stock-Yogo 10% critical value (16.38). Although the urban subsample showed a marginally weak first-stage *F* (8.73), Kleibergen-Paap LM statistics rejected under-identification in all samples (*p* < 0.01).

Second-stage IV estimates robustly confirm that digital literacy significantly increases both exercise frequency and duration, thereby quantitatively addressing Research Question (i). The effects are substantially larger for rural residents (Frequency: +2.05 vs. + 0.99; Duration: +29.34 vs. + 19.23), reinforcing OLS findings and directly resolving Research Question (ii) on urban–rural differentials. Critically, significant negative coefficient on the *digital literacy × urban/rural* interaction term (*p* < 0.01) empirically demonstrates digital literacy’s compensatory role in reducing exercise disparities, providing actionable evidence for targeted policy interventions as envisioned in Research Question (iii).

Second-stage IV estimates consistently demonstrated significant positive effects of digital literacy on exercise frequency and duration (*p* < 0.01), corroborating baseline findings for Research Question (i). Effect sizes remained substantially larger for rural residents (frequency: +2.05 vs. urban +0.99; duration: +29.34 vs. +19.23 min), and the significantly negative interaction term (*p* < 0.01) confirmed digital literacy’s compensatory role in reducing urban–rural disparities (Research Question ii). These results provide empirical grounding for targeted digital empowerment policies (Research Question iii).

#### 3.3.2 Robustness checks

Three supplementary analyses affirmed result robustness:

(1) Alternative outcome specifications (binary participation/nonlinear models)
(2) Working-age subsample restriction (18–59 years)
(3) Exclusion of centrally administered municipalities.

As detailed in Table 6, digital literacy maintained significant positive effects on exercise outcomes across all specifications (*p* < 0.05), with persistently stronger marginal impacts in rural populations. The frequency-specific interaction term remained significantly negative (*p* < 0.01), though duration interactions showed intermittent insignificance in working-age and non-municipal samples—likely reflecting tighter time constraints among employed adults or reduced inter-regional facility disparities. Crucially, exercise frequency demonstrated greater sensitivity to digital literacy improvements in bridging urban–rural gaps, reinforcing the core conclusion that digital literacy robustly promotes physical activity while mitigating geographical inequities.

**Table 6:** Robustness checks.

Models	Exercise participation			Exercise frequency (young and middle-aged)			Exercise duration (young and middle-aged)			Exercise frequency (non-municipal)			Exercise duration (non-municipal)		
Variables	Urban	Rural	Full	Urban	Rural	Full	Urban	Rural	Full	Urban	Rural	Full	Urban	Rural	Full
Digital literacy	1.246^***^	2.277^***^	2.253^***^	1.063^***^	2.467^***^	2.627^***^	23.02^***^	30.10^***^	30.86^***^	1.262^***^	2.031^***^	2.232^***^	28.30^***^	21.40^***^	29.04^***^
	(0.274)	(0.184)	(0.171)	(0.341)	(0.199)	(0.189)	(5.225)	(2.959)	(2.848)	(0.333)	(0.195)	(0.183)	(2.801)	(4.886)	(2.651)
Interaction term			−0.879^***^			−1.791^***^			−7.887			−1.266^***^			−6.649
			(0.238)			(0.303)			(4.819)			(0.280)			(4.154)
Controls	Yes	Yes	Yes	Yes	Yes	Yes	Yes	Yes	Yes	Yes	Yes	Yes	Yes	Yes	Yes
Regional effects	Yes	Yes	Yes	Yes	Yes	Yes	Yes	Yes	Yes	Yes	Yes	Yes	Yes	Yes	Yes
_cons	−4.800^***^	−2.872^***^	−3.655^***^	−3.898^***^	−0.822^***^	−2.034^***^	−53.08^***^	−7.541^**^	−22.39^***^	−4.437^***^	−0.536	−1.784^***^	−4.659	−56.37^***^	−19.61^***^
	(0.538)	(0.298)	(0.256)	(0.575)	(0.285)	(0.252)	(9.087)	(3.811)	(3.532)	(0.585)	(0.282)	(0.250)	(4.032)	(9.023)	(3.629)
N	3,591	9,967	13,558	2,785	7,897	10,682	2,785	7,897	10,682	3,047	12,746	9,699	3,047	9,699	12,746
*r* ^2^	0.078	0.080	0.111	0.098	0.074	0.117	0.091	0.098	0.128	0.095	0.092	0.069	0.082	0.046	0.102

*p < 0.1; **p < 0.05; ***p < 0.01.

## 4 Discussion

This study establishes a significant positive association between digital literacy and physical exercise participation among Chinese urban and rural residents. Quantitatively, a one-unit increase in digital literacy corresponds to a 1.72-unit rise in exercise frequency (*p* < 0.01) and a 26.19-min extension in session duration (*p* < 0.01). Notably, this relationship exhibits marked urban–rural heterogeneity: digital literacy elicits substantially stronger behavioral responsiveness in rural populations, with marginal effects on exercise frequency doubling those in urban areas (+2.05 vs. +0.99 units) and duration increases exceeding urban gains by 53% (+29.34 vs. +19.23 min). Robust to interaction models, IV-2SLS estimation, and sensitivity analyses, these findings lend empirical support to the compensatory advantage hypothesis in health inequality research (35, 36).

### 4.1 Digital literacy as a resource-substitution tool in rural contexts

The heightened efficacy of digital literacy in rural settings aligns with Resource Substitution Theory (RST), which posits that marginalized populations derive greater marginal utility from novel resources when traditional assets are scarce (4). In rural China—where structural deficiencies (e.g., limited sports facilities, socioeconomic constraints, cultural barriers, and low health awareness) impede physical activity (9, 15–17, 37)—digital literacy functions as a catalytic bridge through two compensatory pathways:

*Technology-mediated resource substitution* (Figure 1) enables rural residents to leverage mobile fitness apps (Keep, WeChat Sports, Fitime) and online platforms (Bilibili, Douyin) for structured exercise regimens, effectively compensating for scarce professional trainers, gym infrastructure, and formal physical education. Empirical studies confirm that digitally literate rural older adults are significantly more likely to use fitness apps and seek exercise information online, overcoming spatial constraints (35). Critically, digital home-based programs demonstrate efficacy comparable to in-person training for improving functional capacity, body composition, and aerobic fitness in resource-limited settings (38, 39). However, meta-analytical evidence indicates digital interventions fail among low-SES individuals without adequate digital competencies (4), underscoring digital literacy’s role as the essential enabler that transforms passive access into active health engagement.

*Cognitive-behavioral empowerment* operates through sequential mediation: digital literacy facilitates health information access → enhances health literacy → bolsters self-efficacy → increases physical activity (10, 12). Grounded in Bandura’s social cognitive theory (25), this mechanism is empirically validated by chain-mediation models showing internet use improves self-rated health via self-efficacy and exercise (40). In rural China, survey data reveal digital literacy promotes health behaviors (e.g., exercise, dietary regulation) primarily through happiness and future expectations (41). Notably, rural older adults gain significantly more health literacy per unit of short-video platform use (Douyin, Bilibili) than urban counterparts (42), while credible social media influencers positively shape exercise attitudes and behavioral control (43). Collectively, digital literacy emerges as a substitutive cognitive resource that empowers rural populations to transcend structural disadvantages and adopt sustainable health behaviors.

### 4.2 Policy recommendations

The findings emphasize the crucial role of digital literacy in increasing physical exercise participation, especially among rural residents, where its marginal effect is considerably greater. However, the persistent structural constraints and contextual barriers imply that infrastructure expansion alone is not enough to bridge the urban–rural divide. In line with China’s Digital Village Strategy (18) and National Fitness Plan (2021–2025) (44), a phased, capability-oriented approach is required, going beyond mere infrastructure expansion.

First, the integration of digital literacy training into rural public health and fitness programs is essential. Empirical evidence from this study and prior research suggests that rural residents require targeted skill development to transform passive access into active utilization of health-promoting technologies (45, 46). Digital literacy modules could be embedded into existing community health outreach activities, rural sports festivals, and older adults care services, ensuring that content is adapted to local needs (2, 3)—for example, by using short instructional videos for home-based exercises or agricultural off-season fitness routines. Adopting a “function-first” training model, where digital skills are linked to tangible, valued outcomes such as applying for rural entrepreneurship programs, accessing telehealth services, or joining local online fitness challenges, may enhance engagement. Moreover, leveraging culturally embedded exercise practices such as tai chi or baduanjin and demonstrating their delivery via mobile apps or live-streamed sessions could further increase participation, capitalizing on the high adherence rates observed in similar contexts in low- and middle-income countries (47).

Second, the establishment of community-based digital–physical activity hubs could serve as an effective mechanism to integrate physical infrastructure, digital tools, and social support (48). These hubs should provide safe walking trails, multipurpose courts, and open exercise zones, while also incorporating QR-coded exercise guides, app-linked attendance tracking, and localized health content. Social networks within these hubs—such as volunteer-led exercise groups, peer mentoring, and intergenerational learning programs—can enhance engagement, with digitally literate youth assisting older adults in using fitness apps and wearable devices. Partnerships with local telecom providers could ensure sustained connectivity and affordable device access, following the sustainable integration principles demonstrated in global low-resource digital health initiatives (46).

Third, sustained engagement requires incentive mechanisms that are contextually relevant. Small-scale, app-based reward schemes, where points earned from consistent exercise logging can be redeemed at local shops, could be implemented. Additionally, supporting local influencers and micro-broadcasters to produce credible and relatable fitness content in local dialects may enhance trust and cultural resonance (47). Aligning such incentives with rural seasonal rhythms—for example, initiating post-harvest fitness challenges—could further optimize participation by synchronizing initiatives with residents’ availability and physical readiness.

Fourth, embedding robust monitoring and evaluation systems within rural digital fitness initiatives is vital to avoid “pilot fatigue” and to ensure scalability (46). Standardized metrics for digital literacy and exercise participation could be integrated into rural health surveys and linked with longitudinal frameworks such as CFPS (13, 14). Feedback loops that allow user data to inform iterative improvements to digital fitness tools and community activities would enhance program relevance and sustainability. Furthermore, cross-provincial knowledge-sharing platforms could be established to disseminate best practices and cost-effective intervention models among rural counties.

Finally, addressing the structural barriers to digital inclusion is a prerequisite for impact (4, 45). This includes subsidizing low-cost smartphones or wearable devices preloaded with curated exercise and health applications, particularly for older adults and low-income households. Expanding rural-specific app ecosystems that integrate physical activity promotion with agricultural advisory services, market information, and social networking could help embed exercise into daily digital routines. Additionally, incorporating digital safety and data privacy education into all initiatives would help build trust and promote long-term engagement.

In summary, bridging the urban–rural divide in physical exercise participation requires moving beyond the “access-only” paradigm toward a capability-oriented model. By combining skill development, community-based engagement, structural support, and culturally adapted content, policymakers can unlock the compensatory potential of digital literacy and foster equitable, sustainable health behaviors across China’s diverse geographic and socioeconomic landscapes.

### 4.3 Limitations and future directions

Despite demonstrating the positive effect of digital literacy on physical activity participation, this study faces several limitations. First, due to data constraints, the analysis relies on cross-sectional survey data; future research could employ longitudinal or experimental designs to strengthen causal inference. Second, the measurement of digital literacy may be relatively simplistic, failing to capture the full range of individuals’ digital competencies in diverse scenarios; subsequent research should adopt more nuanced and multidimensional assessment tools or performance-based measures. Third, this study primarily focuses on the Chinese context, and the generalizability of the findings to other cultural or socioeconomic settings remains to be tested. Finally, while prior studies have established links between digital health literacy and health behaviors, some inconsistencies persist across different populations. Future research should therefore explore specific strategies and interventions to enhance digital health literacy in various demographic groups and health contexts.

## 5 Conclusion

This study highlights the significant positive association between digital literacy and physical exercise participation among Chinese residents, with a more pronounced effect observed in rural populations. Enhanced digital literacy correlates with increased exercise frequency and duration, suggesting that digital competencies can effectively promote healthier lifestyles. Notably, the stronger impact in rural areas indicates that digital literacy may serve as a compensatory mechanism, mitigating structural disadvantages and bridging the urban–rural health behavior gap.

These findings underscore the importance of integrating digital literacy initiatives into public health strategies, particularly in underserved rural communities. Policymakers should consider investing in digital infrastructure and education to empower individuals with the necessary skills to access and utilize health-related information and resources. Such efforts could play a crucial role in reducing health disparities and promoting equitable health outcomes across diverse populations.

Future research should explore longitudinal effects and the potential of digital literacy interventions to sustain long-term health behavior changes. Additionally, examining the interplay between digital literacy and other social determinants of health could provide a more comprehensive understanding of the pathways through which digital competencies influence health behaviors.

## Data Availability

The datasets presented in this study can be found in online repositories. The names of the repository/repositories and accession number(s) can be found at: https://cfpsdata.pku.edu.cn/#/resource-detail/4.

## References

[1] Feng Z Zeng Y. Research on accelerating the high-quality development of national fitness empowered by digitalization under the background of building a strong sports country. J Sports Sci. (2023) 43:14–23. doi: 10.16469/j.css.202304002

[2] Liu W Fan B. How digitalization empowers innovation in public service governance for national fitness: an analysis based on Jiaxing practice. J Wuhan Inst Phys Educ. (2025) 59:10–8. doi: 10.15930/j.cnki.wtxb.2025.02.003

[3] Ju L Yang T Gou Y Liu Z Wang Y. Mechanism, challenges, and pathways of digital technology empowering precise supply of sports public services. J Guangzhou Inst Phys Educ. (2025), 45:58–67. doi: 10.13830/j.cnki.cn44-1129/g8.2025.03.006

[4] Western MJ Armstrong MEG Islam I Morgan K Jones UF Kelson MJ. The effectiveness of digital interventions for increasing physical activity in individuals of low socioeconomic status: a systematic review and meta-analysis. Int J Behav Nutr Phys Act. (2021) 18:148. doi: 10.1186/s12966-021-01218-4, PMID: 34753490 PMC8576797

[5] Ji M Deng D Yang X. Influence of internet usage on physical activity participation among Chinese residents: evidence from 2017 China general social survey. Front Public Health. (2024) 12:1293698. doi: 10.3389/fpubh.2024.1293698, PMID: 38873316 PMC11169592

[6] Woessner M Tacey A Levinger-Limor A Parker A Levinger P levinger I. The evolution of technology and physical inactivity: the good, the bad, and the way forward. Front Public Health. (2021) 9:655491. doi: 10.3389/fpubh.2021.655491 34123989 PMC8193221

[7] Dahlgren A Sjöblom L Eke H Bonn SE Trolle Lagerros Y. Screen time and physical activity in children and adolescents aged 10–15 years. PLoS One. (2021) 16:e0254255. doi: 10.1371/journal.pone.0254255, PMID: 34242329 PMC8270173

[8] Kim G Jeong H Yim HW. Associations between digital media use and lack of physical exercise among middle-school adolescents in Korea. Epidemiol Health. (2023) 45:e2023012. doi: 10.4178/epih.e2023012, PMID: 36652903 PMC10581895

[9] van Deursen AJ Dijk JA. The first-level digital divide shifts from inequalities in physical access to inequalities in material access. New Media Soc. (2019) 21:354–75. doi: 10.1177/1461444818797082, PMID: 30886536 PMC6380454

[10] Berkowsky RW Czaja SJ. 2 - challenges associated with online health information seeking among older adults In: Pak R McLaughlin AC, editors. Aging, technology and health. San Diego, CA: Academic Press (2018)

[11] Norman CD Skinner HA. Ehealth literacy: essential skills for consumer health in a networked world. J Med Internet Res. (2006) 8:e506. doi: 10.2196/jmir.8.2.e9 PMC1550701 16867972

[12] Kim H Xie B. Health literacy in the eHealth era: a systematic review of the literature. Patient Educ Couns. (2017) 100:1073–82. doi: 10.1016/j.pec.2017.01.015, PMID: 28174067

[13] Latulippe K Hamel C Giroux D. Social health inequalities and eHealth: a literature review with qualitative synthesis of theoretical and empirical studies. J Med Internet Res. (2017) 19:e6731. doi: 10.2196/jmir.6731 PMC5427250 28450271

[14] Stellefson M Paige SR Alber JM Chaney BH Chaney D Apperson A. Association between health literacy, electronic health literacy, disease-specific knowledge, and health-related quality of life among adults with chronic obstructive pulmonary disease: cross-sectional study. J Med Internet Res. (2019) 21:e12165. doi: 10.2196/12165, PMID: 31172962 PMC6592488

[15] Zhang J Li D Gao J. Health disparities between the rural and urban elderly in China: a cross-sectional study. Int J Environ Res Public Health. (2021) 18:8056. doi: 10.3390/ijerph18158056, PMID: 34360346 PMC8345474

[16] Chang Y. The digital divide between urban and rural areas: characterization, causes, and bridging strategies. Agric Econ Manag. (2025):45–54.

[17] Zhang J Tan Z Tan Q. Research on digital empowerment of rural public sports service governance modernization: a case study of ancient villages in Xiang, Yu, and Qian border regions. J Sports Sci Res. (2025). doi: 10.15877/j.cnki.nsic.20250417.001

[18] Xinhua News Agency. The general office of the CPC central committee and the general office of the State Council issued the digital rural development strategy outline China Government Network (2019). Available online at: https://www.gov.cn/zhengce/2019-05/16/content_5392269.htm (Accessed August 9, 2025)

[19] Ministry of Industry and Information Technology of China. Reply to Proposal No. 5179 of the Second Session of the 14th National People's Congress. Available online at: https://www.miit.gov.cn/zwgk/jytafwgk/art/2024/art_ce1b4f1039844cdc9fd3e76678e3c3e1.html (Accessed May 7, 2025)

[20] Wang F Wang Y. Historical evolution, governance dilemma, and bridging path of China's urban-rural digital divide. China Circ Econ. (2024) 38:3–12. doi: 10.14089/j.cnki.cn11-3664/f.2024.02.001

[21] Ji C Dai J Chen B. Dual differentiation in the era of social media: the urban-rural digital divide in public discourse. Glob Media J. (2024) 11:32–57. doi: 10.26599/GJMS.2024.9330063

[22] Xu Z Xue Z Sun L Hu R. How to promote national participation in physical exercise in China: based on the "elements-environment-technology" analytical framework. J Guangzhou Inst Phys Educ. (2024) 44:30–41. doi: 10.13830/j.cnki.cn44-1129/g8.2024.06.004

[23] Lu X Miao X. Internal mechanisms and changing trends of urban-rural differences in Chinese residents' physical exercise (2010–2021). J Beijing Sport Univ. (2024) 47:70–84. doi: 10.19582/j.cnki.11-3785/g8.2024.03.006

[24] Buja A Rabensteiner A Sperotto M Grotto G Bertoncello C Cocchio S. Health literacy and physical activity: a systematic review. J Phys Act Health. (2020) 17:1259–74. doi: 10.1123/jpah.2020-0161, PMID: 33129198

[25] Bandura A. Social cognitive theory: an agentic perspective. Annu Rev Psychol. (2001) 52:1–26. doi: 10.1146/annurev.psych.52.1.1, PMID: 11148297

[26] China Family Panel Studies (CFPS) Data. (2025). Publish with CFPS data. Available online at: http://www.isss.pku.edu.cn/cfps/en/faq/PublishwithCFPSData/index.htm (Accessed August 8, 2025)

[27] Zou Z Yun Y Sun J. Entropy method for determination of weight of evaluating indicators in fuzzy synthetic evaluation for water quality assessment. J Environ Sci. (2006) 18:1020–3. doi: 10.1016/S1001-0742(06)60032-6, PMID: 17278765

[28] Li X Wang K Liu L Xin J Yang H Gao C. Application of the entropy weight and TOPSIS method in safety evaluation of coal mines. Proc Eng. (2011) 26:2085–91. doi: 10.1016/j.proeng.2011.11.2410

[29] Zhang X Chen W. Does grandchild care intention, intergenerational support have an impact on the health of older adults in China? A quantitative study of CFPS data. Front Public Health. (2023) 11:1186798. doi: 10.3389/fpubh.2023.1186798, PMID: 37693722 PMC10484214

[30] Adler NE Epel ES Castellazzo G Ickovics JR. Relationship of subjective and objective social status with psychological and physiological functioning: preliminary data in healthy, white women. Health Psychol. (2000) 19:586–92. doi: 10.1037/0278-6133.19.6.586, PMID: 11129362

[31] Singh-Manoux A Marmot MG Adler NE. Does subjective social status predict health and change in health status better than objective status? Psychosom Med. (2005) 67:855–61. doi: 10.1097/01.psy.0000188434.52941.a0, PMID: 16314589

[32] Zhou J Guo W Ren H. Subjective social status and health among older adults in China: the longitudinal mediating role of social trust. BMC Public Health. (2023) 23:630. doi: 10.1186/s12889-023-15523-z, PMID: 37013502 PMC10068244

[33] Jiang L He Y Hu C. Public hospital reform, family health consumption and health inequality: evidence from China family panel studies. Front Public Health. (2024) 12:1352417. doi: 10.3389/fpubh.2024.1352417, PMID: 38957205 PMC11217177

[34] Staiger D Stock JH. Instrumental variables regression with weak instruments. Econometrica. (1994) 65:557–86.

[35] Ji H Dong J Pan W Yu Y. Associations between digital literacy, health literacy, and digital health behaviors among rural residents: evidence from Zhejiang, China. Int J Equity Health. (2024) 23:68. doi: 10.1186/s12939-024-02150-2, PMID: 38594723 PMC11003150

[36] Woodley of Menie MA Sarraf MA Peñaherrera-Aguirre M Rindermann H. Parent-offspring resemblance for educational attainment reduces with increased social class in a global sample: evidence for the compensatory advantage hypothesis. Front Psychol. (2024) 14:1289109. doi: 10.3389/fpsyg.2023.1289109, PMID: 38235275 PMC10792003

[37] Al-Worafi YM. Exercises in developing countries: challenges and recommendations In: Handbook of medical and health sciences in developing countries. Cham: Springer (2024)

[38] Langeard A Bigot L Maffiuletti NA Moussay S Sesboüé B Quarck G. Non-inferiority of a home-based videoconference physical training program in comparison with the same program administered face-to-face in healthy older adults: the MOTION randomised controlled trial. Age Ageing. (2022) 51:afac059. doi: 10.1093/ageing/afac059, PMID: 35290431

[39] Physical rehabilitation using telemedicine. J Telemed Telecare. (2007) 13:217–20. doi: 10.1258/135763307781458886 17697506

[40] Liao W Ma C Liu X Sun Z. The chain mediation role of self-efficacy, health literacy, and physical exercise in the relationship between internet use and older adults’ health: cross-sectional questionnaire study. J Med Internet Res. (2025) 27:e73242. doi: 10.2196/73242, PMID: 40591934 PMC12264443

[41] Li R Shao J Gao D. The impact of digital literacy on the health behavior of rural older adults: evidence from China. BMC Public Health. (2025) 25:919. doi: 10.1186/s12889-025-21964-5, PMID: 40055684 PMC11889888

[42] Yu Y Wu Y Huang Z Sun X. Associations between media use, self-efficacy, and health literacy among Chinese rural and urban elderly: a moderated mediation model. Front Public Health. (2023) 11:1104904. doi: 10.3389/fpubh.2023.1104904, PMID: 36969672 PMC10034173

[43] Wang L Li X Wang D Zhu J. Influence of social media fitness influencers’ credibility on users’ physical activity intentions. Digit health. (2024) 10:20552076241302016. doi: 10.1177/20552076241302016, PMID: 39687522 PMC11648026

[44] General Office of the State Council of the People's Republic of China. Notice on issuing the National Fitness Plan (2021–2025). National Sports Administration (2021) Available online at: https://www.sport.gov.cn/gdnps/html/zhengce/content.jsp?id=25531540 (Accessed August 9, 2025)

[45] Liu Y Lachman ME. A group-based walking study to enhance physical activity among older adults: the role of social engagement. Res Aging. (2021) 43:368–77. doi: 10.1177/0164027520963613, PMID: 33021146 PMC8021609

[46] McCool J Dobson R Muinga N Paton C Pagliari C Agawal S. Factors influencing the sustainability of digital health interventions in low-resource settings: lessons from five countries. J Glob Health. (2020) 10:20396. doi: 10.7189/jogh.10.020396 PMC7696238 33274059

[47] Ominyi J Clifton A Cushen-Brewster N. Long-term effectiveness of physical activity interventions for adults across income contexts: a systematic review of strategies and outcome. Bull Fac Phys Ther. (2024) 29:90. doi: 10.1186/s43161-024-00257-9

[48] Gui X Chen Y Caldeira C Xiao D Chen Y. When fitness meets social networks: investigating fitness tracking and social practices on WeRun. Proceedings of the 2017 CHI Conference on Human Factors in Computing Systems. CHI ‘17. New York, NY: Association for Computing Machinery.

