# Supplementary material for: Bridging the urban–rural divide: digital literacy as a catalyst for enhancing physical exercise participation in China
Source: Front Public Health. 2025 Sep 23;13:1630850. doi: 10.3389/fpubh.2025.1630850 (PMC12500683; doi:10.3389/fpubh.2025.1630850)
Supplement: Supplementary file 1 [file Data_Sheet_1.pdf]

# Appendix A: Digital-literacy Index Construction

## 1. Measurement Framework of Digital Literacy Indicators

The digital literacy index was constructed using 12 items from the 2022 China Family Panel Studies (CFPS), grouped into five dimensions. Each item was normalized and weighted using Shannon's entropy algorithm. The table below details the measurement items, scoring rules, and entropy weights:

**Table A1:Digital Literacy Measurement Framework**

| Dimension | Measurement Item | Scoring Rules | Weight |
|---|---|---|---|
| Digital Usage | Combined duration of mobile and computer internet usage (hours/week) | Sum of qu201a (mobile) and qu202a (computer); missing values (-8) set to 0; winsorized at 98th percentile | 0.097 |
| Digital Learning | 1. Importance of internet for learning | qu954: 1 (very unimportant) to 5 (very important); missing values (-8) set to 0 | 0.042 |
| | 2. Online learning behavior | qu94 (yes=1, no=0) and qu941 (daily=2, occasional=1); Composite: Daily=2, occasional=1, no=0 | 0.200 |
| Digital Social | 1. Importance of internet for family/friend contact | qu953: 1 (very unimportant) to 5 (very important); missing values (-8) set to 0 | 0.038 |
| | 2. Use of WeChat | qu11: yes=1, no=0; missing values (-8) set to 0 | 0.039 |
| | 3. Frequency of sharing on WeChat Moments | qu111: 1 (never) to 7 (almost daily); reverse-coded (8-q111); missing values (-8) set to 7 | 0.113 |
| Digital Work | Importance of internet for work | qu951: 1 (very unimportant) to 5 (very important); missing values (-8) set to 0 | 0.045 |
| Digital Life | 1. Importance of internet for daily life | qu955: 1 (very unimportant) to 5 (very important); missing values (-8) set to 0 | 0.045 |
| | 2. Importance of internet for leisure/entertainment | qu952: 1 (very unimportant) to 5 (very important); missing values (-8) set to 0 | 0.043 |
| | 3. Online shopping | qu92 (yes=1, no=0) and qu921 (daily=2, occasional=1); Composite: Daily=2, occasional=1, no=0 | 0.119 |
| | 4. Watching short videos | qu93 (yes=1, no=0) and qu931 (daily=2, occasional=1); Composite: Daily=2, occasional=1, no=0 | 0.015 |
| | 5. Online gaming | qu91 (yes=1, no=0) and qu911 (daily=2, occasional=1); Composite: Daily=2, occasional=1, no=0 | 0.204 |

## 2. Entropy Weight Method: Computational Steps

The entropy weight method objectively assigns weights based on the variability of each indicator. Higher variability implies greater informational value and thus a higher weight. Steps are as follows:

### Step 1: Normalization

For each of the 12 items $x_i$, min-max normalization was applied to rescale values to [0,1]:

$$sx_i = \frac{x_i - \min(x_i)}{\max(x_i) - \min(x_i)}$$

Values of 0 were replaced with 0.0001 to avoid undefined logarithms.

### Step 2: Calculate Probability $p_i$

For each observation j of item $i$:

$$p_{ij} = \frac{sx_{ij}}{\sum_{j=1}^{n} sx_{ij}} \quad (n = sample\ size)$$

### Step 3: Compute Entropy $e_i$

$$e_i = -\frac{1}{\ln(n)} \sum_{j=i}^{n} p_{ij} \ln(p_{ij})$$

### Step 4: Calculate Information Utility $d_i$

$$d_i = 1 - e_i$$

### Step 5: Determine Weight $w_i$

$$w_i = \frac{d_i}{\sum_{i=1}^{12} d_i}$$

### Step 6: Composite Score

The digital literacy index for each individual is the weighted sum:

$$Digital\ Literacy\ Score = \sum_{i=1}^{12} w_i \cdot sx_i$$

## 3. Stata Implementation Code

### Step 1: Normalization and winsorization

```
global items x1 x2 x3 x4 x5 x6 x7 x8 x9 x10 x11 x12
foreach var in $items {
    sum `var', detail
    gen s`var' = (`var' - r(min)) / (r(max) - r(min))
    replace s`var' = 0.0001 if s`var' == 0
}
```

### Step 2: Probability matrix

```
forval i = 1/12 {
    egen sum_sx`i' = sum(sx`i')
    gen p`i' = sx`i' / sum_sx`i'
}
```

### Step 3: Entropy calculation

```
forval i = 1/12 {
    gen temp`i' = p`i' * ln(p`i')
    egen sum_temp`i' = sum(temp`i')
    gen e`i' = -sum_temp`i' / ln(18336)    // n=18,336
    gen d`i' = 1 - e`i'
}
```

### Step 4: Entropy weights

```
egen total_d = rowtotal(d1-d12)
forval i = 1/12 {
    gen w`i' = d`i' / total_d
}
```

### Step 5: Composite score

```
forval i = 1/12 {
    gen score_part`i' = w`i' * sx`i'
}
egen digital_literacy = rowtotal(score_part1-score_part12)
```

## 4. Validation

**Weights**: Sum to 1.000 (validation: =0.097+0.042+⋯ +0.204=1).

**Index Range**: 0 (lowest) to 1 (highest digital literacy).

**Sensitivity**: Weights are data-driven, reflecting each indicator’s discriminative power.
